# Supplementary material for: Causal Relationship between Aging and Anorexia Nervosa: A White-Matter-Microstructure-Mediated Mendelian Randomization Analysis
Source: Biomedicines. 2024 Aug 16;12(8):1874. doi: 10.3390/biomedicines12081874 (PMC11351342; doi:10.3390/biomedicines12081874)
Supplement: Supplementary file 1 [file biomedicines-12-01874-s001.zip › Supplementary Figure/Figure S4/Singe SNP plot of MR analysis for TL to ubm-b-1506.pdf]

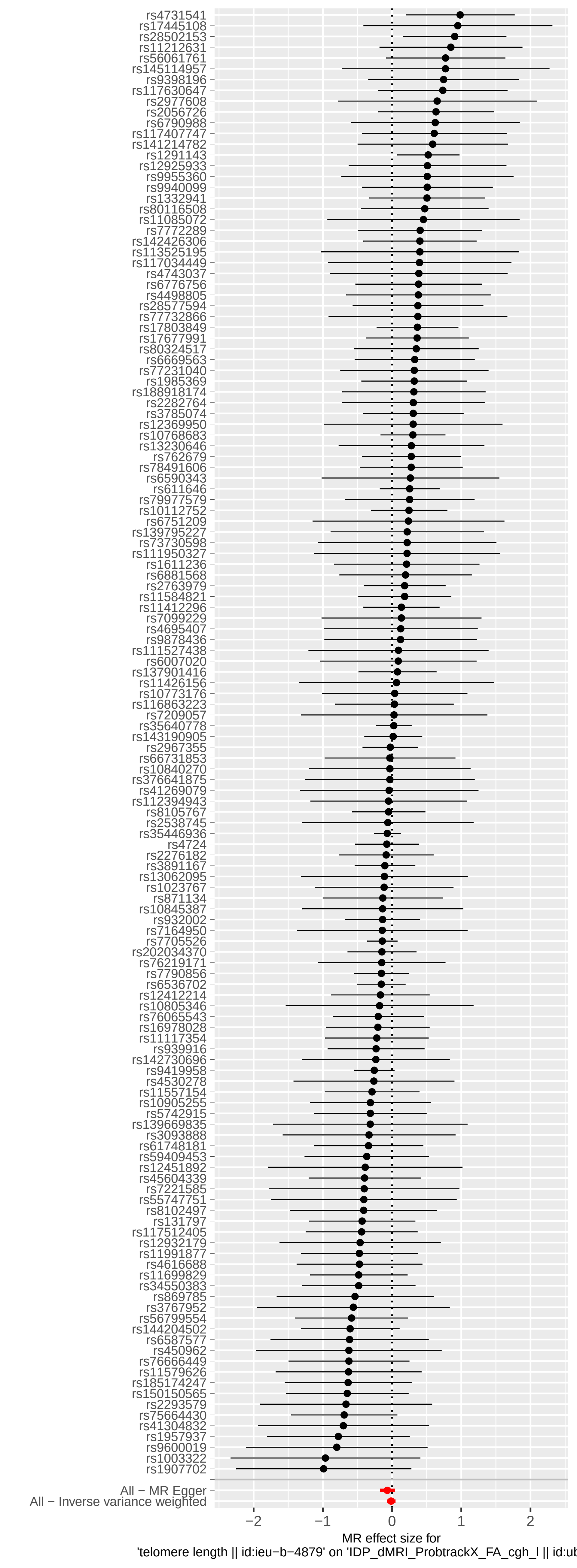
rs4731541
rs17445108
rs28502153
rs11212631
rs56061761
rs145114957
rs9398196
rs117630647
rs2977608
rs2056726
rs6790988
rs117407747
rs141214782
rs1291143
rs12925933
rs9955360
rs9940099
rs1332941
rs80116508
rs11085072
rs7772289
rs142426306
rs113525195
rs117034449
rs4743037
rs6776756
rs4498805
rs28577594
rs77732866
rs17803849
rs17677991
rs80324517
rs6669563
rs77231040
rs1985369
rs188918174
rs2282764
rs3785074
rs12369950
rs10768683
rs13230646
rs762679
rs78491606
rs6590343
rs611646
rs79977579
rs10112752
rs6751209
rs139795227
rs73730598
rs111950327
rs1611236
rs6881568
rs2763979
rs11584821
rs11412296
rs7099229
rs4695407
rs9878436
rs111527438
rs6007020
rs137901416
rs11426156
rs10773176
rs116863223
rs7209057
rs35640778
rs143190905
rs2967355
rs66731853
rs10840270
rs376641875
rs41269079
rs112394943
rs8105767
rs2538745
rs35446936
rs4724
rs2276182
rs3891167
rs13062095
rs1023767
rs871134
rs10845387
rs932002
rs7164950
rs7705526
rs202034370
rs76219171
rs7790856
rs6536702
rs12412214
rs10805346
rs76065543
rs16978028
rs11117354
rs939916
rs142730696
rs9419958
rs4530278
rs11557154
rs10905255
rs5742915
rs139669835
rs3093888
rs61748181
rs59409453
rs12451892
rs45604339
rs7221585
rs55747751
rs8102497
rs131797
rs117512405
rs12932179
rs11991877
rs4616688
rs11699829
rs34550383
rs869785
rs3767952
rs56799554
rs144204502
rs6587577
rs450962
rs76666449
rs11579626
rs185174247
rs150150565
rs2293579
rs75664430
rs41304832
rs1957937
rs9600019
rs1003322
rs1907702
All − MR Egger
All − Inverse variance weighted
−2
−1
0
1
2
MR effect size for
'telomere length || id:ieu−b−4879' on 'IDP_dMRI_ProbtrackX_FA_cgh_l || id:ub
